# The Course and Variation of the Facial Vein in the Face—Known and Unknown Facts: An Anatomical Study

**DOI:** 10.3390/medicina59081479

**Published:** 2023-08-17

**Authors:** Martin Siwetz, Hannes Widni-Pajank, Niels Hammer, Ulrike Pilsl, Simon Bruneder, Andreas Wree, Veronica Antipova

**Affiliations:** 1 Division of Macroscopic and Clinical Anatomy, Gottfried Schatz Research Center, Medical University of Graz, Auenbruggerplatz 25, A-8036 Graz, Austria; 2 Department of Oral and Maxillofacial Surgery, Klagenfurt Am Wörthersee Clinic, Feschnigstraße 11, A-9020 Klagenfurt am Wörthersee, Austria; 3 Department of Orthopedic and Trauma Surgery, University of Leipzig, D-04103 Leipzig, Germany; 4 Division of Biomechatronics, Fraunhofer Institute for Machine Tools and Forming Technology Dresden, D-09126 Dresden, Germany; 5 Department of Oral and Maxillofacial Surgery, Medical University of Graz, Auenbruggerplatz 5, A-8036 Graz, Austria; 6 Institute of Anatomy, Rostock University Medical Center, Gertrudenstr. 9, D-18057 Rostock, Germany

**Keywords:** anatomical variation, extracranial venous system, clinical significance facial anatomy, facial vein

## Abstract

*Background and Objectives*: The facial vein is the main collector of venous blood from the face. It plays an important role in physiological as well as pathological context. However, to date, only limited data on the course and tributaries of the facial vein are present in contemporary literature. The aim of this study was to provide detail on the course and the tributaries of the facial vein. *Materials and Methods*: In 96 sides of 53 body donors, latex was injected into the facial vein. Dissection was carried out and the facial vein and its tributaries (angular vein, ophthalmic vein, nasal veins, labial veins, palpebral veins, buccal and masseteric veins) were assessed. *Results*: The facial vein presented a textbook-like course in all cases and crossed the margin of the mandible anterior to the masseter in 6.8% of cases, while being located deep to the zygomaticus major muscle in all cases and deep to the zygomaticus minor in 94.6% of cases. *Conclusions*: This work offers detailed information on the course of the facial vein in relation to neighboring structures, which shows a relatively consistent pattern, as well as on its tributaries, which show a high variability.

## 1. Introduction

An in-depth understanding of the vascular anatomy of the face plays an important role in clinical practice including planning for surgical procedures in the face, such as free flaps, as well as in increasingly popular procedures such as filler or botulinum toxin injections. In all procedures, knowledge of the vascular anatomy is essential both to improve the results and to minimize the risk of complications [1,2,3,4,5,6]. There is an abundance of literature on the facial artery, its course, branching and variations [7,8,9,10,11,12,13,14,15,16,17,18,19,20,21,22,23,24]. Much less is known about the anatomy of the facial vein, and its role in blood drainage as well as a boundary structure for fat compartments [13,25,26,27,28]. In this regard, the facial vein has previously been shown to form the lateral border of deep medial buccal fat, the lateral border of the premaxillary space (which contains the deep nasolabial fat compartment), and the medial border of the suborbicularis oculi fat [28,29,30,31,32,33]. Therefore, the facial vein plays a pivotal role as a hallmark structure of the face. It is of importance for understanding the detailed anatomy of the facial fat compartments. Complications such as an irreversible blindness resulting from filler or autologous fat transfer procedures could be avoided taking anatomy of the veins of the face into account [25,34]. Moreover, the facial artery and vein have been used as one of the stems for face transplants and were even demonstrated to be able to support a highly complex composite facial allograft [35,36,37,38]. The facial vein, also referred to as the anterior facial vein, is a paired vessel and the main venous drainage for the blood of the facial region [27]. It is formed by the confluence of two roots, namely the superficial root being the angular vein and the deep root being the profound vein of the face. The latter communicates with the pterygoid plexus and takes a course as the angular vein from the medial angle of the eye to the anterior portion of the masseter muscle then further drains towards the internal jugular vein [25,39,40,41]. Tributaries of the facial vein in the facial region include the angular vein, which forms the origin and provides drainage for the supraorbital and supratrochlear veins, the lateral vein of the nose and the dorsal vein of the nose who both provide drainage for the blood of the nasal region, the inferior palpebral veins, the superior and inferior labial vein, the profound vein of the face, as well as the buccal and masseteric veins [13,25,39,42,43]. To date, limited data on the course and tributaries of the facial vein exist. The aim of this study was to provide detailed information on the course of the facial vein in relation to neighboring structures, as well as on its tributaries and their variability.

## 2. Materials and Methods

For this given study, 96 half faces from 53 individuals were included. The body donors included 23 males and 30 females at an age at time of death of 39–96 years. All corpses were embalmed using a modified Thiel technique [44,45]. While alive, all body donors had given their informed consent for the donation of their postmortem tissues for research purposes. All body donors were bequeathed to the Division of Macroscopic and Clinical Anatomy of the Medical University of Graz (Austria) under the approval of the Anatomical Donation Program of the Medical University of Graz and in accordance with the Austrian laws concerning body donations. 

Body donors were only included in this study if they showed no major pathological lesions, former surgeries, or tumors in the facial region. For better visibility of the smaller tributaries of the facial vessels as well as for better discrimination between small arteries and small veins, the main trunk of each vessel was injected with colored latex. For the facial vein, blue-colored latex was used, the injection mass consisted of 70% distilled water and 30% nature-latex GIVUL MR (Fa. Helmut Bergk, Frankfurt/Main, Germany). This injection mass was mixed with blue color for venous injection, while for the facial artery, the same injection mass was mixed with red color.

### 2.1. Dissection

The skin incision was performed from the midline along the margin of the mandible to the angle of the mandible and then cranially 2 cm anterior to the auricle. Beginning from the lateral, a skin flap was elevated medially. The injected facial vein was located at the inferior margin of the mandible and traced upwards opposite its flow direction. Dissection was carried out along the main stem and each smaller vessel taking care not to cut any tributaries. All tributaries of the facial vein were characterized and documented based on their presence, course, numbers and branching pattern, and topography to the mimic musculature.

### 2.2. Exclusion Criteria

Vessels and vessel tributaries were only included in data evaluation if the condition of the tissues allowed for a data acquisition without potential errors. Therefore, any half faces in which the vessel tributaries were not adequately filled with latex had to be excluded for the specific assessment.

## 3. Results

### 3.1. Course of the Facial Vein 

**Figure 1:**
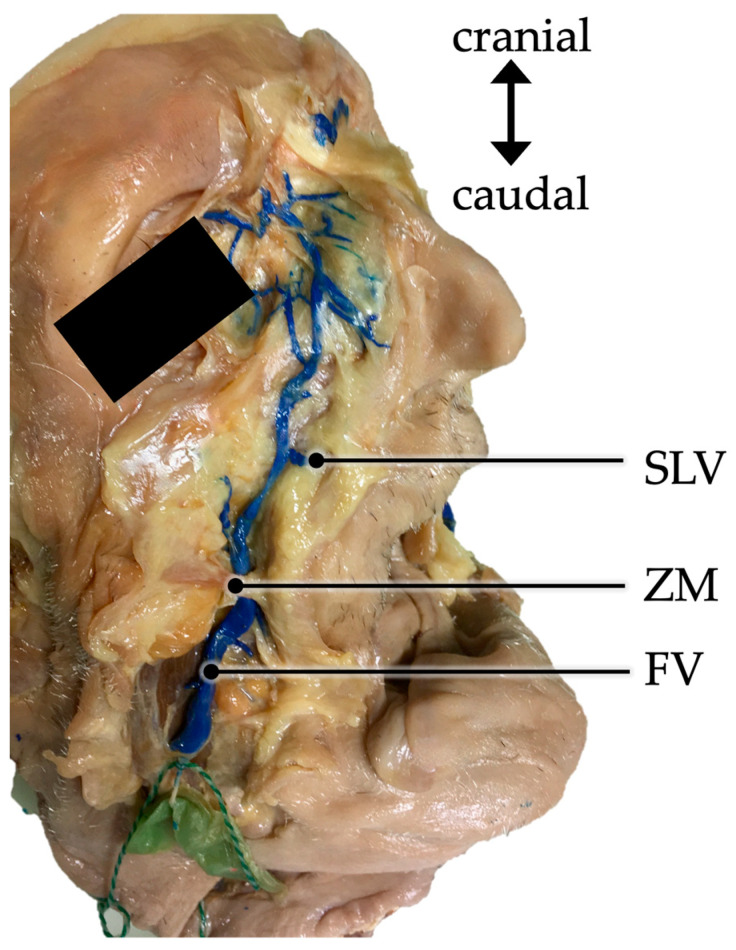
Right hemiface of a male body donor. The facial vein (FV) is injected with blue-colored latex and dissected. It is taking a course from the medial angle of the eye towards the mandible passing below the zygomatic muscles (ZM). SLV— superior labial vein.

The facial vein in all cases took a textbook-like course in, which after its origin as the angular vein, it took a straight or slightly curved course in the latero-caudal direction above the zygomatic process of the maxilla towards the margin of the mandible (Figure 1). Its relation to the facial muscles as well as its branching is described in the following.

### 3.2. Relation to the Masseter and Zygomatic Muscles

**Figure 2:**
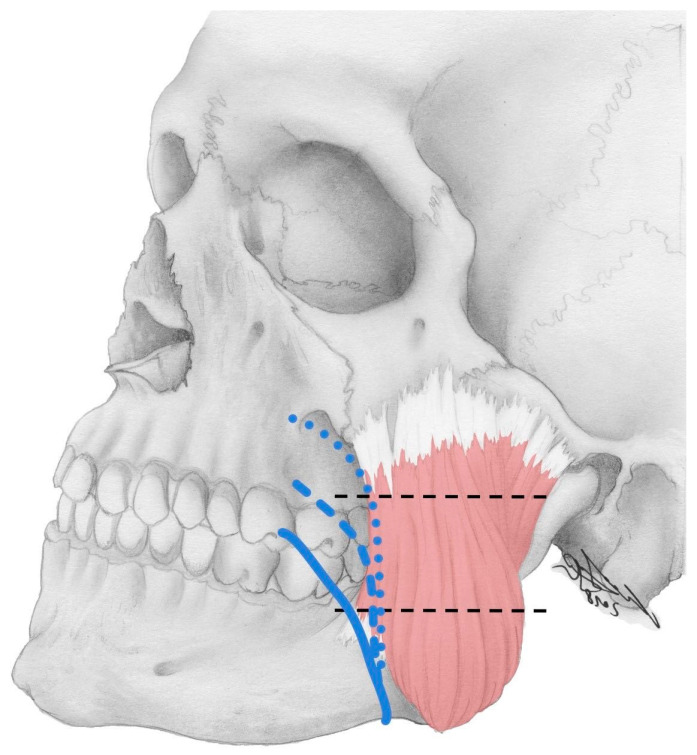
The course of the facial vein with regard to the masseter muscle is depicted. In 6.8% of cases, the vein was located anterior to the masseter at the base of the mandible (not shown). In 71.9% (full line) it crossed the anteriormost fibers of the masseter in its lower third, in 23.2% (dashed line) in the middle third and in 4.9% (dotted line) in the upper third. The horizontal dashed lines mark the lower, middle and upper third of the masseter muscle.

Along with the tributaries of the facial vein, its relation to the masseter muscle was assessed in 88 cases. In 6.8% (6/88) of cases, the facial vein was located in front of the masseter muscle, even at the base of the mandible. In the remaining 93.2% (82/88) of cases, at the base of the mandible, the facial vein was located behind the most anterior fibers of the masseter muscle. During their course, 59 of these veins crossed the masseter and its anterior border in its lower third, 19 in its middle third and 4 in its upper third (Figure 2). 

**Figure 3:**
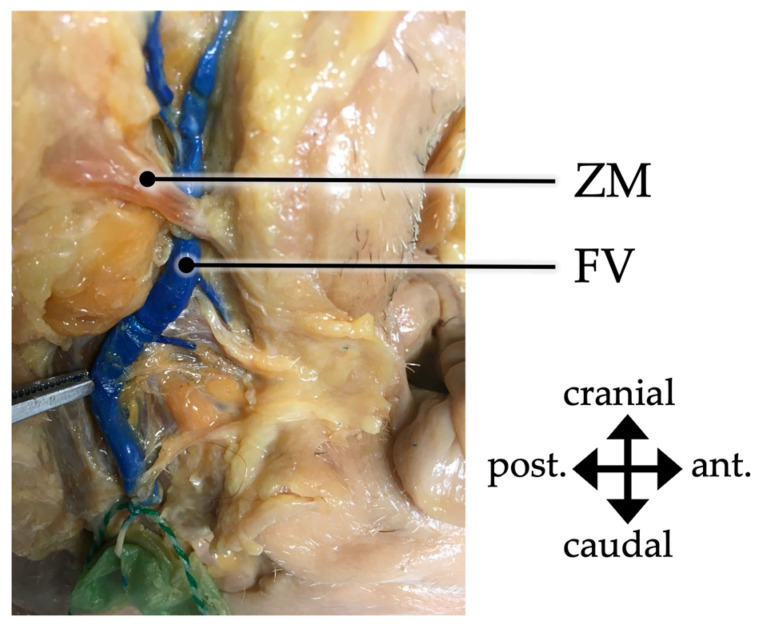
Detailed depiction of the facial vein (FV) passing deep to the zygomatic muscles (ZM) on the right side of a male body donor. post.—posterior; ant.—anterior.

The relation of the facial vein to the zygomaticus major muscle could be assessed in 52 of cases. In all of these cases the facial vein was located deep to the muscle. The relation to the zygomaticus minor muscle could be assessed in 37 cases. In 94.6% (35/37) of cases, the facial vein was located deep to the muscle (Figure 3) while only in 5.4% (2/37), the vein was located superficial to the muscle. 

### 3.3. Supraorbital and Supratrochlear Vein

**Figure 4:**
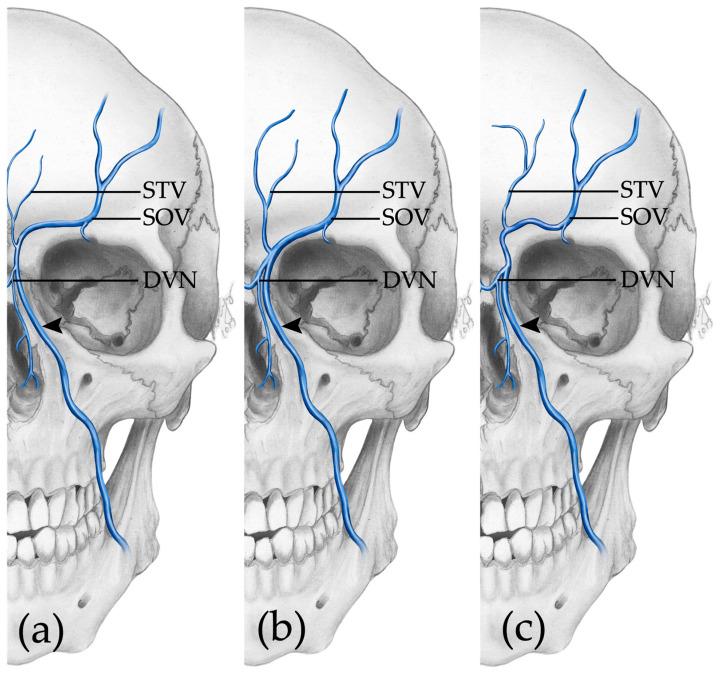
Types of drainage of the supraorbital and supratrochlear veins. (**a**) Type A: Drainage into the angular vein at or above the level of the dorsal vein of the nose. (**b**) Type B: Drainage into the angular vein below the level of the dorsal vein of the nose. (**c**) Type C: Drainage into the supratrochlear vein. ➤—angular vein; STV—supratrochlear vein; SOV—supraorbital vein; DVN—dorsal vein of the nose.

The supraorbital vein was assessed in 59 of 96 half faces. The remaining 47 half faces were excluded due to the mentioned criteria. According to the pattern of drainage three types of supraorbital veins could be observed:

- Type A: Drainage into the angular vein at or above the level of the dorsal vein of the nose (Figure 4a);
- Type B: Drainage into the angular vein below the level of the dorsal vein of the nose (Figure 4b);
- Type C: Drainage into the supratrochlear vein (Figure 4c).

Type A was found in 86.4% (51/59) of cases, while in this group, in 29 cases, the drainage into the angular vein happened alongside the supratrochlear vein. Type B was present in 5.08% (3/59) of cases. Type C was present in 8.5% (5/59) cases; the supraorbital vein drained into the supratrochlear vein, which then drained into the angular vein.

### 3.4. Venous Drainage of the Frontal Region

Dissection of the frontal region showed interesting results as, on one hand, anastomoses between the right and left sides of the face were commonly present with a pronounced ring-shaped anastomotic system in the frontal region, which was found in 12 faces. Alternatively, a characteristic pattern of drainage was formed including the supratrochlear, supraorbital and dorsal vein of the nose which are further described below. 

### 3.5. Angular Vein

**Figure 5:**
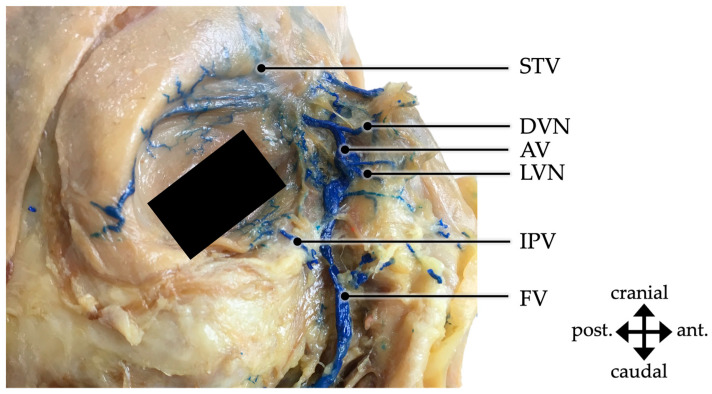
Right facial region of a female body donor. The facial vein (FV) is injected with blue latex color and is taking a course from superior–medial to inferior–lateral with its tributaries being, among others, the supratrochlear vein (STV), dorsal vein of the nose (DVN), angular vein (AV), lateral vein of the nose (LVN) and inferior palpebral vein (IPN). post.—posterior; ant.—anterior.

The angular vein was assessed in 91 of the 96 half faces, while five half faces were excluded. The angular vein was present in a textbook-like fashion before the orifice of the superior labial vein in 98.9% (90/91) (Figure 5). In 1.1% (1/91) of cases, an angular vein was not present. In that case, the facial vein was originating through an orifice of the inferior palpebral vein and the superior labial vein, while the superior ophthalmic vein was draining towards the contralateral side, therefore resulting in an anastomosis with the contralateral facial vein.

### 3.6. Ophthalmic Veins

In the 91 cases in which the angular vein was assessed, the ophthalmic veins were assessed as well. In 80.2% (73/91) of cases, a superior ophthalmic vein was present and could be traced towards the orbita. In 47.3% (43/91) of cases, an inferior ophthalmic vein was present. 

### 3.7. Veins of the Nose

The nasal veins, being the dorsal vein of the nose and the lateral vein of the nose, were assessed in 84 of the 96 half faces. Twelve had to be excluded due to the mentioned criteria. The dorsal vein of the nose was present in 85.7% (72/84) of cases. In all these cases, it took a course from the medial angle of the eye, where an anastomosis with the angular vein was present and took a course to the root of the nose and along the dorsum of the nose. 

At least one lateral nasal vein was present in 96.4% (81/84) of cases; in 3.6% (3/84) of cases, it was not present. However, in all of these three cases, a dorsal vein of the nose was present. In 19.1% (16/84) of cases, a single lateral vein of the nose was present, which then formed an anastomosis with the angular vein. In 42.9% (36/84) of cases, there were two lateral veins of the nose present, which in their course either formed an anastomosis with the dorsal vein of the nose (in five cases) or with the angular vein. In 25% (21/84) of cases, three lateral veins of the nose where present. In four cases one of these veins formed an anastomosis with the dorsal vein of the nose, while in all other cases, they formed an anastomosis with the angular vein. Four or more lateral veins of the nose were present in 9.5% (8/84) of cases (Table 1). The veins of the nose can be seen in Figure 5. 

### 3.8. Labial Veins

The superior labial vein was assessed in 89 of 96 cases. A superior labial vein was present in 100% (89/89) of hemifaces. Regarding its course, in 97.8% (87/89) of cases, it took an upwards-directed course to orifice into the facial vein at the level of the ala of the nose or slightly below. In 2.3% (2/89) of cases, the course was directed downward to the angle of the mouth; thereof in one case, a common trunk with the inferior labial vein before orifice into the facial vein was present. 

Anastomoses to the contralateral side of the face in the region of the upper lip were present in 10.4% (10/89) of cases. Furthermore, in 9% (8/89) of cases, an accessory smaller vein parallel to the superior labial vein was present. 

The inferior labial vein was assessed in 93 of 96 cases. An inferior labial vein was present in 84.8% (79/93) of cases. Its further path of drainage was the facial vein in all cases, but in one case, a common trunk with the superior labial artery was present. Its orifice into the facial vein was at the level of the lower row of teeth. 

### 3.9. Palpebral Veins

**Figure 6:**
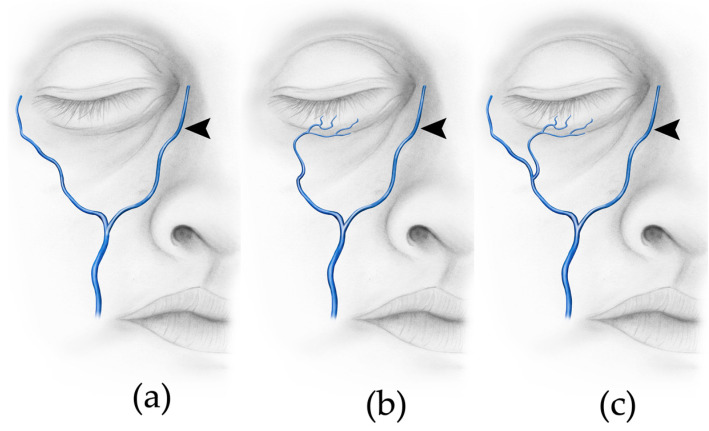
Overview of the palpebral veins. In 50% of cases (**a**), a vein from the lateral margin of the orbita was present. In 24% (**b**), a vein located at the middle of the lower eyelid was present, and in 6.3% (**c**), both of the above-mentioned veins were present. ➤ marks the angular vein.

Palpebral veins were assessed in all 96 cases. In 80.2% (77/96) of cases, at least one palpebral vein was present. In 50% (48/96) of cases, this referred to a vein originating at the lateral margin of the orbita and draining into the facial vein below the superior labial vein. In 24% (23/96) of cases, this referred to a vein that originated approximately in the middle of the lower eyelid and also drained into the facial vein below the superior labial vein (Figure 5). In 6.3% (6/96) of cases, both the above-described veins were present. In three cases, they drained into the facial vein together in the same place, but without prior formation of a common trunk, and in three cases, they drained separately. An overview can be found in Figure 6.

### 3.10. Deep Facial Vein

In 54 of 96 cases (56.3%), a connecting vein between the pterygoid plexus and the facial vein—in the manner of a deep facial vein—was present. In 20 of these 54 cases (37%), further tributaries parallel to the main deep facial vein were present. The orifice was very variable and showed no specific patterns. Regarding the topography, the deep facial vein and its accompanying tributaries were located superficial to the buccinator and deep to the masseter muscle. 

### 3.11. Buccal and Masseteric Veins

In 72.9% (70/96) of hemifaces, veins to the soft tissues and musculature of the cheek were present. Overall, there were 77 masseteric veins and 132 buccal veins present (Table 2). These veins all had their orifice into the facial vein caudal to the superior labial vein, but in relation to the inferior labial vein, 54.6% of the masseteric and 16.7% of the buccal veins drained into the facial vein below the inferior labial vein. 

## 4. Discussion

Most reports on the vascular anatomy of the face so far focused on the facial artery and its course, variations and tributaries. However, in a majority of these studies, the facial vein is omitted [9,12,21,46,47]. Its course is often described in the literature as being rather straight and superficial from the medial canthus towards the anterior margin of the masseter muscle [40,43,48]. Only few studies provided the results using anatomical dissection [13,25]. Up to date, no publications showed the schematic detailed variations of the course and patterns of a facial vein in a way they did for the facial artery [7,8,9,10,12,13,14,17,18,19,21,22,23,24]. This given study described, for the first time in detail, not only the variations in the course but also the variations of all tributaries and all inflows of the facial vein. 

### 4.1. The Facial Vein Shows a Consistent Course through the Face

In the literature, especially in anatomy textbooks, the course of the facial artery is described consistently as being straight from the medial angle of the eye towards the anterior portion of the masseter muscle [40,43,48]. Moreover, the course and the patterns of the facial vein are described as being quite predictable, only with rare variations ranging between 0.3 and 2 percent [13,25,49]. However, there are authors describing consistent communications between the facial vein and other veins of the face, being the superior ophthalmic and temporal vein [50]. 

Lohn and colleagues described the facial vein as a vessel, that in almost all cases was located dorsal to the facial artery and took a rather straight course towards the canthus [13]. Also, Houseman et al. [51] and Zhou et al. [49] likewise described the course of the facial artery straight from the medial corner of the eye to the lower edge of the lower jaw. Therefore, it can be assumed, that the facial vein was taking a course slightly dorsal to the artery. Cotofana and colleagues further quantified the location and course of the facial vein. They found it to cross the margin of the mandible at the anterior margin of the masseter muscle with an overlap of 0.2–1.0 cm [25]. These findings can be backed by our findings of the facial vein crossing the margin of the mandible anterior to, or at the anteriormost fibers of the masseter muscle. 

### 4.2. Dorsal Location to the Anterior Most Fibers of the Masseter Muscle

Molinari et al. [52] examined the course of the facial vein and found that it runs in front of the masseter muscle and can also cross its foremost muscle fibers in order to run further caudally on its surface.

Cotofana et al. [25] described that the facial vein in the area of the lower edge of the mandible was always (100%) behind the foremost fibers of the insertion of the masseter muscle, at a distance between 0.2 and 1.0 cm.

In the present study, an average distance of 3.20 mm from the facial vein to the anterior fibers of the insertion of the masseter muscle was found. On the one hand, the result of Cotofana et al. was confirmed. Furthermore, the facial vein was located deep to the zygomaticus major muscle in all cases and deep to the zygomaticus minor muscle in 94.6% of cases.

### 4.3. Supraorbital, Supratrochlear and Angular Vein—Three Types Can Be Described

Regarding the tributaries of the facial vein, only very few reports are present in the literature. The supraorbital and supratrochlear veins are described as draining their blood towards the angular vein [42,48]. Cotofana and colleagues found the supratrochlear and supraorbital vein drained into the angular vein; however, there are no further subtypes described by these authors [25]. Based on our findings and based on the level of drainage as well as the direction of drainage, we can describe three types of supraorbital veins for the first time (Figure 4). 

The angular vein is described as forming the origin of the facial vein. It drains the blood of the supraorbital and the supratrochlear vein and takes course downwards profound to the orbicularis oculi muscle and superficial to the levator labii superioris alaeque nasi muscle [39,42]. In a more recent study, Cotofana and colleagues found the angular vein coursing superficial to the levator labii superioris alaeque nasi muscle but deep to the orbicularis oculi muscle in all cases, analogue to the results of older literature [40,43,48]. Furthermore, they found the angular vein draining the external nasal vein in all cases [25]. 

### 4.4. Veins of the Nose Were Present in Almost All Cases

At least one lateral nasal vein was present in 96.4% of cases and a dorsal vein of the nose was present in 85.7% of cases. If present, the dorsal vein of the nose took a course from the medial angle of the eye along the dorsum of the nose towards the root of the nose. The number of lateral nasal veins was quite variable ranging from zero to four and more veins. This can be backed by Cotofana and colleagues, who found external nasal veins to be given off by the angular vein in 100% of cases [25]. An anastomotic venous connection between the right and left hemiface could be described by Cotofana et al. at the level of the root of the nose [25]. This anastomotic connection was then further described as the transverse nasal root vein by Shimizu and colleagues [53]. 

### 4.5. Labial Veins Showed a Consistent Course

The course we found of the superior labial vein, being slightly cranial towards the nose and to the orifice into the facial vein at the level of the ala of the nose or slightly below in 97.8% of cases can be backed by Yamamoto and colleagues, who found the superior labial vein taking a horizontal course to drain into the facial artery at the level of the lateral side of the ala of the nose [54]. 

### 4.6. Palpebral Veins—Three Types Can Be Described

Lee and colleagues assessed the venous drainage of the lower eyelid and determined four categories of inferior palpebral veins, similar to our types. In 22.0%, they found the inferior palpebral vein to be underdeveloped, which is similar to the 19.9% in which we did not find any inferior palpebral vein. Most commonly, they found a single vein originating from the lateral margin of the orbita in 58% of cases. A single vein from the middle of the eyelid, inferior to the orbicularis oculi, was found in 12%, and a combination of both veins in 8% [55]. This is similar to our findings, where we found a vein from the lateral angle of the eye in 50% of cases as compared to 58%, a vein from the middle of the eyelid in 24% of cases as compared to 12% and a combination in 6.3% of cases as compared to 8%. Differences, however, may be explained in different sample sizes or even ethnics. 

### 4.7. Buccal Veins, Masseteric Veins and Deep Facial Vein—Common Tributaries

Overall, 77 masseteric and 132 buccal veins present draining into the facial vein below the superior labial vein and in variable fashion in regard to the inferior labial vein. Furthermore, a connection between the pterygoid plexus and the facial vein, being the deep facial vein, was present in 54 cases, with no specific pattern regarding the level of the orifice into the facial vein. To our knowledge, no studies by now have described the number or pattern of the buccal and masseteric veins. 

### 4.8. Clinical Significance

The understanding of the vascular anatomy of the face, including the facial vein and its tributaries and their given variability and anastomoses, plays an important role in clinical practice. This includes the planning of procedures with increasing popularity such as filler and botulinum toxin injections as well as more complex surgical procedures such as free flaps. Deeper knowledge of vessel anatomy and variations should lead to improved results as well as well as a reduced risk of complications [1,2,3,4,5,6]. Therefore, the results of this study should act as a base for surgical anatomy since veins’ locations and drainage routes have a pivotal role as hallmark structures in the face. Their great importance results from the avoidance of possible complications or as a prerequisite for successful surgical procedures: irreversible blindness can result from filler or autologous fat transfer procedures [25,34], and vessel anatomy is essential in free flaps and even face transplants [35,36,37,38]. Therefore, further studies targeting on the anatomy in regard to specific procedures should be performed. 

## 5. Conclusions

The facial vein shows a relatively consistent pattern regarding its course from the medial angle of the eye towards the margin of the mandible. However, its tributaries are highly variable. 

## Figures and Tables

**Table 1:** Pattern of drainage of the nasal veins.

Number of Lateral Veins of the Nose (Number of Cases)	Drainage into the Angular Vein Number of Veins	Drainage into the Dorsal Vein of the Nose Number of Veins
0 (3)	-	-
1 (16)	16	0
2 (36)	67	5
3 (21)	59	4
≥4 (8)	32	3

**Table 2:** Pattern of numbers of buccal veins, based on their number.

Number of Buccal Veins	Number of Cases
1	30 (43.5%)
2	21 (30.4%)
3	14 (20.3%)
4	2 (2.9%)
5	2(2.9%)

## Data Availability

Not applicable.
